# Single-cell transcriptomics reveal differences between chorionic and basal plate cytotrophoblasts and trophoblast stem cells

**DOI:** 10.21203/rs.3.rs-6797967/v1

**Published:** 2025-06-11

**Authors:** Robert Morey, Francesca Soncin, Sampada Kallol, Nirvay Sah, Zoe Manalo, Tony Bui, Jaroslav Slamecka, Virginia Chu Cheung, Don Pizzo, Daniela F. Requena, Ching-Wen Chang, Omar Farah, Ryan Kittle, Kelly Lam, Morgan Meads, Mariko Horii, Kathleen M. Fisch, Mana M. Parast

**Affiliations:** 1 Department of Pathology, School of Medicine, University of California San Diego, La Jolla, 92093, CA, USA.; 2 Sanford Consortium for Regenerative Medicine, La Jolla, 92093, CA, USA.; 3 Center for Perinatal Discovery, University of California San Diego, La Jolla, 92093, CA, USA.; 4 Department of Obstetrics, Gynecology, and Reproductive Sciences, School of Medicine, University of California San Diego, La Jolla, 92093, CA, USA.

**Keywords:** Placenta, Trophoblast, Cytotrophoblast, Trophoblast stem cells

## Abstract

Cytotrophoblast (CTB) of the early gestation human placenta are bipotent progenitor epithelial cells, which can differentiate into invasive extravillous trophoblast (EVT) and multinucleated syncytiotrophoblast (STB). Trophoblast stem cells (TSC), derived from early first trimester placentae, have also been shown to be bipotential; however, their cell-of-origin has not been identified. In this study, we set out to probe the transcriptional diversity of early and late first trimester villous CTB (vCTB) and compare these to TSC. To this end, we performed single-cell RNA sequencing (scRNA-seq) on placental villous tissue from early (6–8 weeks) and late (12–14 weeks) first trimester placentae; we also evaluated CTB within basal (maternal) and chorionic (fetal) regions of early first trimester placenta, both by scRNA-seq and GeoMx-based spatial transcriptomics. Finally, we performed scRNA-seq on three TSC lines, derived from 6–8 week gestation placentae, as well as on early first trimester CTB at several timepoints during TSC derivation. We found notable distinctions within CTB clusters based on gestational age, further influenced by location near the basal or chorionic plates. We identified trophoblast states representing “initial state” vCTB (*in vivo* CTB progenitors), as well as additional CTB subtypes, precursor STB, and precursor and mature EVT. CTB progenitors were enriched in early first trimester placentae at the basal plate; overall, basal plate CTB were biased toward EVT, and chorionic plate CTB toward STB, precursors. Clustering and trajectory inference analysis indicated that TSC were most like EVT precursor cells. In fact, vCTB lost their *in vivo* “initial state” markers, including PAGE4, as they transitioned to TSC during *in vitro* culture. This was confirmed by flow cytometric analysis of 6 different TSC lines, which showed uniform expression of proximal column markers ITGA2 and ITGA5. Additionally, we found that ITGA5^+^ CTB could be plated in 2D, forming only EVT upon spontaneous differentiation, but failed to form self-renewing organoids; conversely, ITGA5^−^ CTB could not be plated in 2D, but readily formed organoids. Our findings suggest that distinct CTB states exist in different regions of the placenta as early as six weeks gestation and that current TSC lines most closely resemble ITGA5^+^ CTB, biased toward the EVT lineage.

## 1 Introduction

The development of the human placenta has been a black box, particularly early in gestation, when the organ is difficult to probe during an ongoing pregnancy[1–3]. Following implantation, the cytotrophoblast (CTB) shell expands and invaginations soon give rise to primary and secondary chorionic villi[4, 5]. Villous CTB (vCTB) progenitor cells differentiate into two main mature trophoblast types: syncytiotrophoblast (STB), which arise by CTB fusion and serve at the nutrient/gas exchange interface of the floating chorionic villi; and extravillous trophoblast (EVT), which arise through epithelial-mesenchymal transition within the anchoring villi, ultimately invading through decidua and the upper third of the uterine myometrium, remodeling maternal spiral arterioles in order to access oxygenated blood for continuous growth and development of the fetus[6]. In 2018, trophoblast stem cells (TSC) were derived from early gestation vCTB, and media formulations developed to allow these cells to either self-renew or differentiate into both EVT and STB[7]. This work significantly advanced our ability to study this early “black box” period of placental development, spawning a wealth of publications on factors and pathways that regulate human trophoblast differentiation[8–13]. However, while these cells are derived from ITGA6^+^ vCTB, it is unclear how well they represent this bipotential cell population, particularly given their lack of spontaneous differentiation into STB.

Recently, Sheridan et al.[14] compared TSCs to trophoblast organoids (T-Org), which can also be derived from early gestation placentae, and are composed of an outer layer of proliferative CTB and an inner layer of spontaneously-formed STB[15, 16]. They found that TSCs have a distinct transcriptome, expressing several markers of cells in the proximal column of anchoring villi, regions which harbor cells transitioning into EVT[14]. Subsequently, Shannon et al. applied single cell RNA-sequencing (scRNA-seq) to compare T-Org and TSC grown in 3D as organoids (TSC-Org) to primary trophoblast within the early gestation placenta and found that TSC-Org show features of both CTB and EVT[17, 18]. Here, we set out to evaluate CTB heterogeneity in first trimester human placenta at the single cell level, first comparing early (6–8 weeks) to late (12–14 weeks) first trimester CTB, and subsequently, identify a bipotential “initial state” CTB, which we find to be enriched within the basal plate (BP, maternal surface), rather than the chorionic plate (CP, fetal surface) of early first trimester placentae. We then use a combination of spatial transcriptomics and scRNA-seq and identify important differences in differentiation potential between CTB in the BP vs. CP regions of first trimester placenta. We then superimpose single cell transcriptomics from TSC, cultured in 2D, onto our *in vivo* dataset, and determine that these cells are distinct from the “initial state” villous CTB (vCTB), and most closely resemble, not vCTB, but proximal column trophoblast, on their way to differentiating into EVTs. Using scRNA-seq and flow cytometry, we note the loss of initial state CTB and gain of EVT progenitor state features during the TSC derivation process. Finally, we confirm by flow cytometry that TSCs uniformly express proximal column trophoblast/EVT progenitor markers, ITGA5 and ITGA2, and show that similar cells isolated from first trimester placentas exclusively differentiate into EVT. We conclude that TSCs represent a unique cell type, resembling only rare cells *in vivo*, and are distinct from bipotential villous CTB in early gestation placentae.

## 2 Results

### 2.1 Transcriptional topography of first trimester trophoblast at single cell resolution revealed heterogeneity of cytotrophoblast based on gestational age

**Fig. 1:**
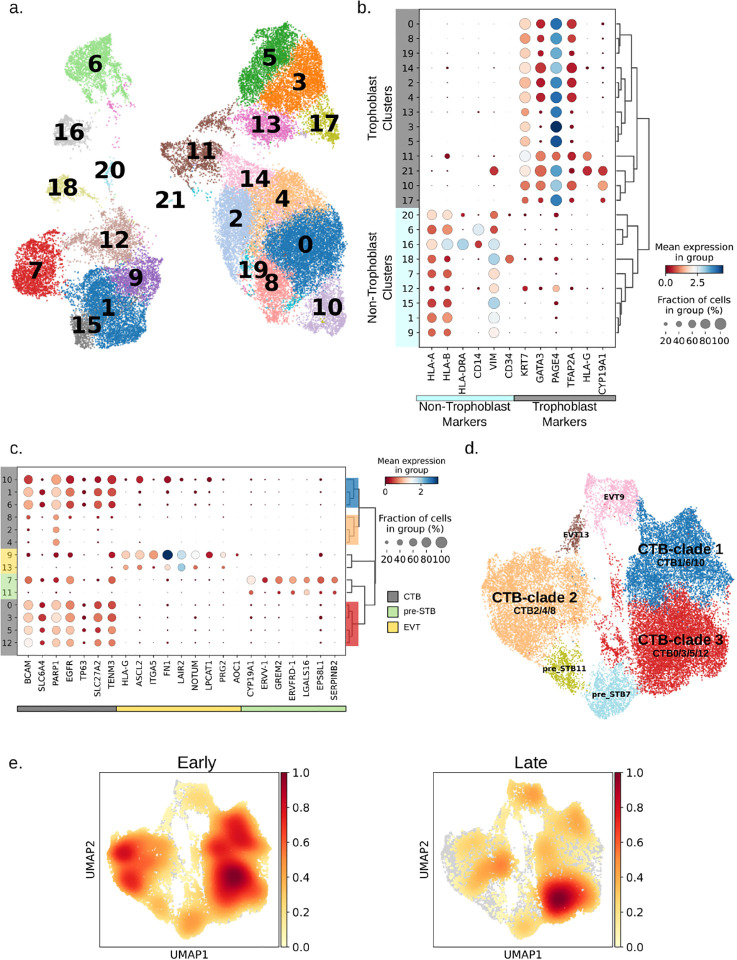
Integrated placental single cell RNA-seq data, showing distinct CTB clades in early vs. late first trimester. (**a**) UMAP of all placental cells following quality control filtering, integration, and clustering. (**b**) Dot plot and hierarchical clustering of cell clusters using trophoblast and non-trophoblast specific gene expression. (**c**) Dot plot and hierarchical clustering of cell clusters using cell type specific gene expression. Dendrogram is colored based on the CTB clade color in the UMAP in part (d). (**d**) UMAP of trophoblast cells annotated based on cell type specific gene expression shown in part (c). Clades were formed based on hierarchical clustering seen in part (c) and in dendrogram in **Fig. S2d**. (**e**) UMAPs depicting the cell density in early (left) and late (right) first trimester placentae.

To better understand the transcriptional landscape of first trimester human trophoblast, we performed single cell RNA-seq on four first trimester normal placentas, two early (6–8 weeks gestational age/GA) and two late (12–14 weeks GA). Only the gestational sac and associated villous tissue (no decidual tissues) were dissociated into single cells. In total, approximately 60,000 cells were captured using the 10X Genomics platform and sequenced (**Table S1**). The samples underwent ambient RNA removal, doublet identification and filtering, as well as cell and gene quality control and filtering (See Methods), resulting in approximately 44,000 cells that were then clustered, integrated, and visualized using uniform manifold approximation and projection (UMAP)[19–22] (**Fig. S1a, S1b**). Leiden clustering resulted in 22 clusters, and based on marker genes, the non-trophoblast cells generally clustering on the left side of the UMAP and the trophoblast cells generally clustering on the right (Fig. 1a). Clusters were annotated as either trophoblast or non-trophoblast in origin using the expression of trophoblast specific genes (such as *KRT7, GATA3, PAGE4, TFAP2A, HLA-G, CYP19A1*) and non-trophoblast genes (such as *HLA-A, HLA-B, HLA-DRA, CD14, VIM, CD34*) (Fig. 1b, **Table S2**). We were interested exclusively in the trophoblast clusters (0, 2, 3, 4, 5, 8, 10, 11, 13, 14, 17, 19) and removed all non-trophoblast clusters (**Fig. S2a**). After the removal of the non-trophoblast clusters, we reclustered the remaining trophoblast cells into 14 clusters and recalculated the marker genes for each cluster (**Fig. S2b, S2c, Table S3**).

Next, we annotated the clusters using a panel of curated marker genes, into three broad trophoblast cell types: 1) cytotrophoblast (CTB) (*BCAM, SLC6A4, PARP1, EGFR, TP63, SLC27A2, TENM3*); 2) extravillous trophoblast (EVT) (*HLA-G, ASCL2, ITGA5, FN1, LAIR2, NOTUM, PRG2, AOC1*); and 3) pre-syncytiotrophoblast (pre-STB) (*CYP19A1, ERVV-1, GREM2, ERVFRD-1, LGALS16, EPS8L1, SERPINB2*). We designated cells expressing canonical syncytiotrophoblast (STB) specific genes as pre-STB because STB are large multinucleated cells that presumably would not be able to be captured by the 10X Genomics Chromium platform. The resulting 14 clusters included ten different CTB clusters, two EVT clusters, and two pre-STB clusters (Fig. 1c, **S2d, Table S3, S4**). Based on hierarchical clustering, the CTB clusters could be combined into three groups\clades (Fig. 1d, **S2d**): CTB-clade 1 (containing clusters CTB1, CTB6, and CTB10) and CTB-clade 2 (containing CTB2, CTB4, and CTB8) were transcriptionally similar, and distinct from CTB-clade 3 (containing clusters CTB0, CTB3, CTB5, and CTB12) (Fig. 1d, **S2d, Table S4**).

To better understand the differences between the cells comprising each CTB clade, we determined the cell density of the early and late first trimester cells (Fig. 1e, **Table S4**) and noted that, while all CTB clades contained early first trimester cells, CTB-clade1 and CTB-clade2 lacked late first trimester cells. We found that our late first trimester-enriched CTB-clade3 expressed 10 genes significantly higher than clades1 and 2. These upregulated genes (*OLR1, VSIR, SLC40A1, SLC22A11, FLT1, CSF3R, KDM5B, FBN1, MALAT1, CCDC18-AS1*) play regulatory roles in the placenta, particularly in angiogenesis, immune regulation, structural integrity, and cellular differentiation, all of which are vital for successful pregnancy and fetal health (**Table S6**). Next, we determined the differences between the CTB clades that contained relatively few late first trimester cells (CTB clades1 and 2). Using genes upregulated in CTB-clade1 by at least a log2 fold change of 1.5, we found that the top five MSigDB Hallmark pathways significantly (adjusted p-value < 0.05) enriched were pathways related to cell proliferation and differentiation (G2-M checkpoint, E2F targets, Myc targets v1, Epithelial Mesenchymal transition/EMT, and Apical junction) (**Table S7**). Genes upregulated in CTB-clade2 were significantly enriched for pathways relating to KRAS signaling and immune response (Allograft rejection, Complement, and Inflammatory Response) (**Table S8**). Notably, although CTB-clade2 did not express any EVT or pre-STB markers and clustered with the other CTB clusters, only a small fraction of cells expressed mature cytotrophoblast markers such as *EGFR, TP63*, and *BCAM*, compared to the other CTB clades (Fig. 1c).

### 2.2 Trophoblast trajectory inference identifies a common progenitor CTB cluster

**Fig. 2:**
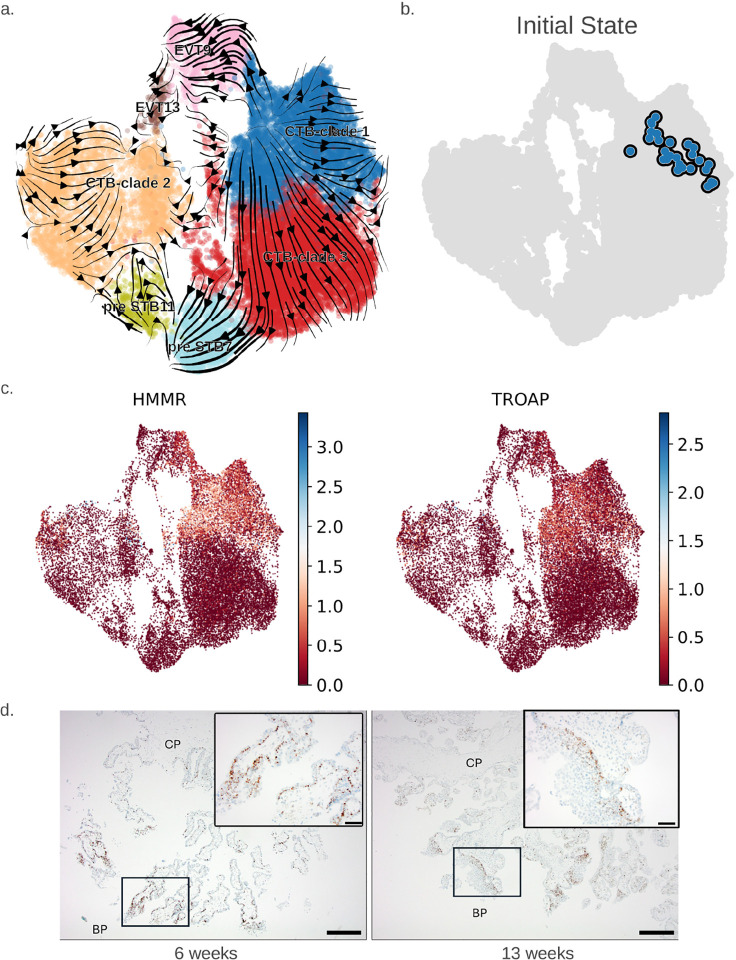
Identification of “initial state” CTB within early first trimester placentae. (**a**) RNA velocity projected as streamlines on the integrated first trimester trophoblast UMAP. CTB names refer to CTB clades after combining CTB clusters in Fig. 1c. (**b**) Initial state cells (n=30) as calculated by CellRank. (**c**) Expression of the initial state markers *HMMR* and *TROAP* on the integrated first trimester trophoblast UMAP. (**d**) *In-situ* hybridization of *HMMR* expression in early and late first trimester placenta. Low-power micrographs show chorionic (CP) and basal (BP) plate regions (scale bar = 625 *μ*m); inset is a magnified micrograph of the BP region (scale bar = 125 *μ*m).

Next, to study the cellular dynamics and relationships between cells and clusters, we performed trajectory inference on our integrated trophoblast cells using RNA velocity[23]. In agreement with previous results, our analysis suggested a common CTB initial state (CTB-clade1) with two differentiation trajectories; one to a terminal EVT (EVT9) cell population and one to a pre-STB (pre-STB7) cluster (Fig. 2a). Through ranking genes based on their cluster-specific differential velocity expression (significantly higher or lower compared to the remaining clusters in the population), we identified genes that best explained the RNA velocity vector field (**Table S9**). We found that the rank velocity genes (r^2^ > 0.5 and *ρ* > 0.3) in CTB-clade1 were significantly enriched in the epithelial mesenchymal transition (EMT) hallmark pathway (adj. p-value < 0.1 × 10^−8^) as well as the Reactome regulation of IGF binding protein pathway (adj. p-value < 0.0005). Additionally, we were interested in marker genes that were differentially expressed in the initial state, which was calculated using CellRank[24, 25] and included only early first trimester cells (Fig. 2b). We therefore looked for genes that were differentially upregulated (adj. p-value < 0.05 and log fold change > 1) in the initial state cells (n=30) compared to the rest of the trophoblast cells in our integrated trophoblast dataset and were part of the CTB1 rank velocity genes (**Table S9**). Not surprisingly, these differentially upregulated initial state genes were predominantly genes related to mitosis, such as *HMMR* and *TROAP* (Fig. 2c), but also included several transcription factors such as *MXD3, CENPA, ZNF302, ZNF165, ZNF701, TET1, KMT2A, KLF6*, and *TEAD1* (**Fig. S3, Table S10**). To identify the location of these initial state cells, we performed *in-situ* hybridization for one of the more highly-expressed markers, *HMMR*, in five early (6 week) and five late (13 week) first trimester placental tissues. We confirmed that these cells are more numerous in early first trimester placentas, and significantly more enriched at the basal, compared to chorionic plate (Fig. 2d).

### 2.3 Spatial transcriptome analysis reveals differences between basal and chorionic plate CTB

The chorionic (CP) and basal (BP) plate constitute the fetal and maternal surfaces of the placenta, respectively, with the chorionic villi emanating from the former and being anchored within the latter. These two surfaces are functionally different but the CTB composition of these two regions has yet to be systematically evaluated. As expected, our initial state cells were predominantly from early first trimester placentas (Fig. 1e, Fig. 2b–c), and more abundant at the basal plate (Fig. 2d). To better understand the composition of the cell types that made up our early first trimester CTB, we used the GeoMx digital spatial profiler, and applied its whole transcriptome atlas (WTA) panel to 3 additional 6-week placentas (**Table S1**), selecting regions of interest (ROI’s) at/near the CP (sac) or BP (anchoring villi) of these placentas, and segmenting on CTB by EGFR immunostaining (**Fig. S4a**). We next performed differential expression analysis (adj. p-value < 0.05 and log fold change > 1) between the BP and CP ROIs (**Fig. S4b, Table S11**). The genes that were upregulated in the BP-CTB were enriched for the MSigDB Hallmark hypoxia and mTORC1 Signaling pathways (adj. p-value < 0.05) whereas the genes that were upregulated in the CP-CTB were enriched for the EMT pathway (adj. p-value < 0.0005) (**Table S11**). We next asked what cells in our integrated early and late first trimester placental scRNA-seq dataset were transcriptionally most similar to our BP vs. CP CTB cells. We therefore scored our scRNA-seq cells using the significantly upregulated BP and CP genes from our spatial dataset. We found that the BP-CTB genes were upregulated in the EVT clusters, and the CP-CTB genes were upregulated in our pre-STB clusters (**Fig. S4c**). These data suggest that there may be important differences in differentiation potential between CTB in the BP and CP regions of first trimester placenta.

**Fig. 3:**
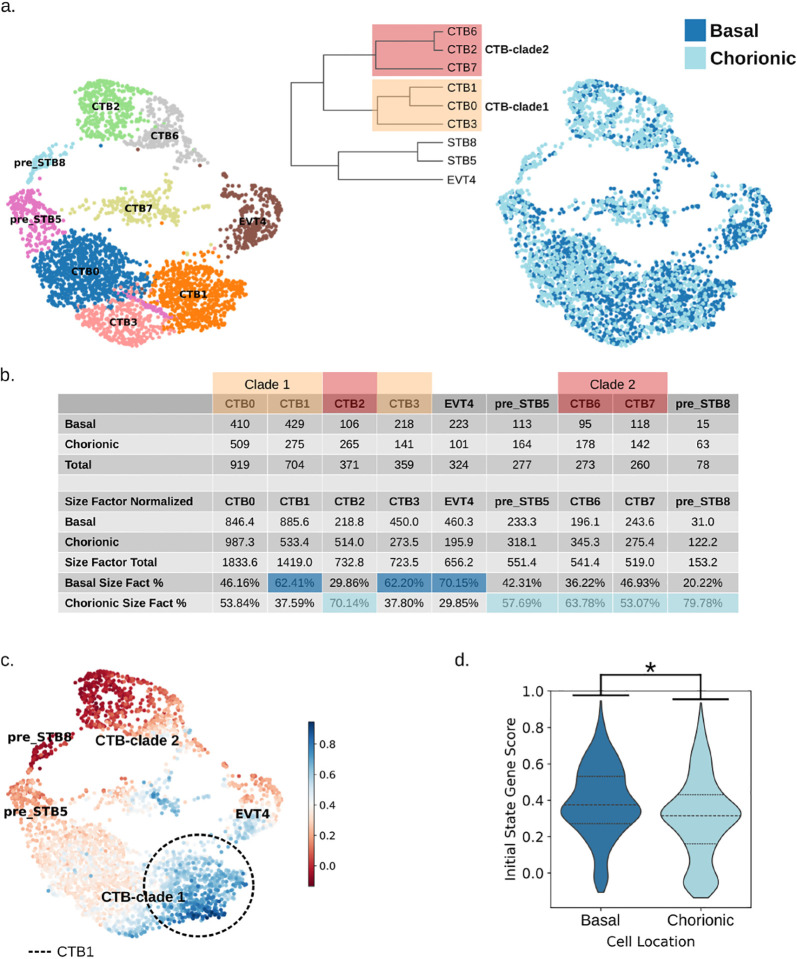
Identification of distinct CTB clades within basal and chorionic plate regions of early first trimester placenta. (**a**) UMAP of annotated and integrated basal and chorionic plate trophoblast from two early first trimester placentae, showing individual clusters (left) and placental region (dark blue for basal plate and light blue for chorionic plate) (right). Dendrogram (middle) shows transcriptomic similarity (Pearson correlation) between UMAP clusters. (**b**) Table showing the number of cells comprising each cluster, including both raw numbers and size factor-normalized, originating from either the basal or chorionic plate. (**c**) Expression of the initial state markers (**Table S10**), in the basal and chorionic plate UMAP. Dashed circle marks cells in cluster CTB1. (**d**) Violin plot showing the initial state score difference between all the basal and chorionic plate cells (Wilcoxon rank-sum test, p-value=1.67e-33 indicated by *). Dashed lines show quartiles.

To better understand the differences between cells in the basal and chorionic plate we dissected the CP (sac) and BP (anchoring villous tips) regions of two early first trimester placentas, isolated CTB from each of these regions using Percoll gradient centrifugation and performed single cell RNA-seq on the resulting four samples (**Table S1**). The capturing of the two compartments was confirmed by enrichment of *CDX2* and *ASCL2* in the CP and BP cells, respectively, by qPCR[26, 27] (**Fig. S5a**). Following integration and removal of non-trophoblast cells (**Fig. S5b**), the data were re-clustered (Fig. 3a), broadly annotated using the same marker genes as with the early and late first trimester whole placenta data (**Fig. S5c**), and the composition source of each cluster was calculated (Fig. 3b). The CTB clusters separated into two clades: CTB-clade1 was composed of clusters CTB0/1/3, and CTB-clade2 was composed of clusters CTB2/6/7 (Fig. 3a). Not surprisingly, the EVT cluster contained a higher percentage of cells from the basal plate and the pre-STB clusters a higher percentage of cells from the chorionic plate (Fig. 3b). Interestingly, after adjusting for the total amounts of basal and chorionic cells in the dataset, CTB-clade2 contained a higher proportion of chorionic cells, whereas CTB-clade1 contained more basal cells (Fig. 3a–b). We next verified that the upregulated basal and chorionic plate genes in our spatial transcriptomic dataset were similarly expressed in our basal and chorionic plate scRNA-seq dataset. We observed that the basal and chorionic CTB exhibited higher BP- and CP-CTB gene score, respectively, although the difference was more pronounced between the cells with the CP-CTB score (Wilcoxon rank-sum p-value < 0.001) (**Fig. S6a, S6b**).

To identify the location of initial state cells in this BP/CP dataset, we scored the dataset using the initial state genes from our early and late first trimester dataset (**Table S10**). We discovered that the differentially expressed genes representing the initial state exhibited the highest expression levels within the CTB1 cluster, which was primarily (62%) composed of basal plate cells (Fig. 3b, 3c). Upon comparing all basal and chorionic cells using these initial state genes, the basal plate cells exhibited significantly higher expression levels of these genes, compared to the chorionic cells (Wilcoxon rank-sum p-value < 1.7 × 10^−23^) (Fig. 3d). Additionally, we evaluated expression of our initial state markers, *HMMR* and *TROAP*, as well as the transcription factors *MXD3* and *CENPA*, shown to be upregulated in our initial state cells (**Fig. S3**), in the BP/CP dataset and noted that these genes are, again, highly expressed in the CTB1 cluster (**Fig. S7a, S7b**). We looked more closely at the localization of the initial state gene, *HMMR*, using *in situ* hybridization of early first trimester placentas, and confirmed enriched expression of this gene in proximal column trophoblast at the basal plate, but also in some CTB at or near the chorionic plate (**Fig. S7c**). These results confirm that initial state CTB are enriched at/near the basal plate, though some also reside at/near the chorionic plate.

### 2.4 TSC show some similarities to initial state CTB but have unique gene expression

**Fig. 4:**
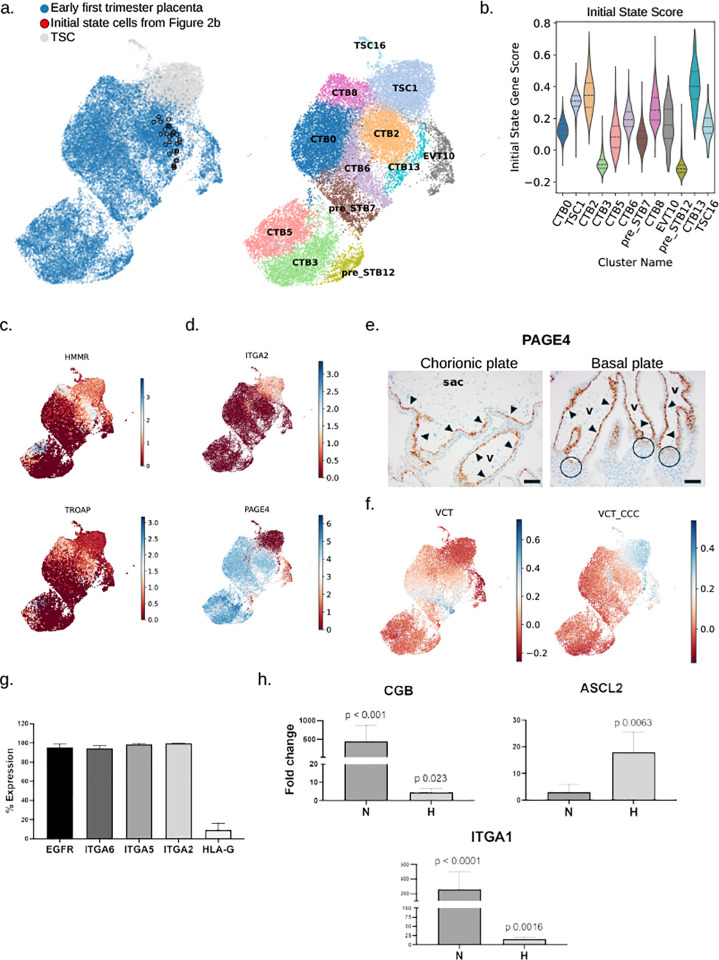
Comparison of TSC to early first trimester trophoblast identifies differences between TSC and initial state CTB. (**a**) UMAP of integrated first trimester trophoblast and TSC, annotated based on cell type specific gene expression, as shown in **Fig. S8c (right)**. Location of initial state cells (n=30) determined in Fig. 2b **(left)** is highlighted using red color and black outline. (**b**) Violin plot showing the initial state score by cluster, based on expression of genes significantly upregulated by initial state cells (Figure 2b, **Table S10**). Dashed lines show quartiles. (**c**) UMAPs showing expression of initial state markers *HMMR* (top) and *TROAP* (bottom). (**d**) UMAPs showing expression of pcEVT marker *ITGA2* (top) and vCTB marker *PAGE4* (bottom). (**e**) *In-situ* hybridization of *PAGE4* in a 6-week placenta, showing uniform expression in villous CTB (arrowheads) within the gestational sac (“sac”), as well as in chorionic villi (“v”) near the chorionic and basal plates, but excluded from proximal column trophoblast (circles). Scale bar=125 *μ*m. (**f**) UMAP visualizations depicting the scores of villous cytotrophoblast (VCT) and cytotrophoblast cell columns derived from VCT (VCT-CCC) cell types based on the top 50 uniquely expressed genes as outlined by Arutyunyan et al[8] (**g**) Percent expression of villous CTB (EGFR and ITGA6), proximal column EVT (ITGA5 and ITGA2), and pan-EVT (HLA-G) markers in undifferentiated TSCs by flow cytometry. Data are reported as mean ± standard deviation of 6 distinct TSC lines. (**h**) Quantitative PCR of STB marker CGB, and EVT markers ASCL2 and ITGA1, in TSC spontaneously differentiated over four days in normoxia (N, 21% oxygen) or hypoxia (H, 2% oxygen). Data are presented as fold change over undifferentiated TSC, reported as mean ± standard deviation (n=2), with p-values calculated using one-way ANOVA.

In 2018, Okae et al. derived trophoblast stem cells (TSC) from ITGA6^+^ CTB fraction of early first trimester (6–8 week gestational age) placentae, and demonstrated their capacity to differentiate into both EVT and STB[7]. Since then, many groups, including ours, have used these or other similarly-derived cell lines, to study various aspects of trophoblast differentiation[28]. We therefore sought to compare these TSCs to various subgroups of early first trimester CTB in order to identify where these stem cells fall along the developmental pseudotemporal order. We used three TSC lines, derived in our lab from early gestation (6–7 week) placentae, and performed scRNA-seq (**Table S1**). Following integration with our early first trimester (6–8 week) placental scRNA-seq data (**Fig. S8a**), we performed clustering and annotated the clusters based on the same markers used to annotate the placental trophoblast clusters (**Fig. S8b, S8c, Table S12**). As TSCs are thought to be similar in function to an initial state CTB, we next identified where in our integrated early first trimester/TSC dataset the previously-determined initial state cells (Fig. 2b) were located (Fig. 4a). Additionally, we scored the cells in our dataset using the significantly upregulated initial state marker genes (**Table S10**) and examined the expression of the previously-identified initial state marker genes *HMMR* and *TROAP*. Our analysis revealed that clusters TSC1 and TSC16, which were predominantly (> 90%) comprised of cells from TSC lines, expressed many CTB markers (**Fig. S8c**), had a moderate initial state gene score (Fig. 4b), and did exhibit some expression of initial state markers (*TROAP* and *HMMR*) (Fig. 4c). However, particularly in the case of TSC1 (the larger of the two TSC clusters), our TSC clusters also expressed many EVT markers (**Fig. S8c**); in fact, clusters TSC1 and TSC16 were transcriptionally most similar to the EVT10 cluster (**Fig. S8d**).

Differential expression analysis between our TSC clusters and the initial state cells revealed striking differences (**Table S13**). For example, the noncanonical genes *SOX15, SOX9*, and *CD9* were significantly more highly expressed (adj. p-value < 1×10^−6^ and log fold change > 4) in the TSC clusters relative to our initial state cells (**Fig. S9a**), as were the proximal column trophoblast/EVT progenitor associated genes *ITGA2* and *ITGA5* (Fig. 4d, **Fig. S9a**; adj. p-value < 1×10^−4^ and log fold change > 26). In contrast, the widely expressed CTB markers, *PAGE4, BCAM, FOXP1, FOXO4, PBX1, PEG3, PEG10, SERPINF1*, and *GPX3*, several of which were identified by Shannon et al as controllers of CTB-specific regulons, were not expressed in our TSC clusters (Fig. 4d, **Fig. S9b**)[18]. We confirmed by immunostaining that, within first trimester trophoblast, ITGA2 and ITGA5 are confined to HLA-G^+^ cells of trophoblast cell columns within anchoring villi, with rare ITGA2^+^ cells identified only within the proximal column, but absent in villous CTB and STB (CYP19A1^+^) (**Fig. S9c, S9d**) as previously reported[18, 29]. We also performed dual *in-situ* hybridization for ITGA5 and ITGA2 in first trimester placentae, and confirmed that rare dual-positive cells are only present within the proximal column (**Fig. S9e**). Additionally, we performed *in-situ* hybridization with a PAGE4-specific probe and validated our scRNA-seq data, showing that this gene has the converse expression pattern as ITGA2 and ITGA5, with expression present only in villous CTB and lacking in trophoblast cell columns (Fig. 4e).

In order to identify the placental cell type with the closest transcriptional resemblance to our TSC, we utilized the top 50 cell type marker genes from Arutyunyan et al.[8] to analyze our integrated early first trimester/TSC dataset. Our analysis revealed that the TSC exhibited the highest similarity to the VCT-CCC cluster (Fig. 4f). These cells, described by Arutyunyan et al.[8] as a proliferative cell type likely originating from the villous cytotrophoblast (VCT) and giving rise to extravillous trophoblasts (EVTs), are otherwise known as proximal column EVT (pcEVT)[30]. These results suggest that TSCs contain characteristics of progenitor state CTB but have distinct transcriptional programs and may lack the same STB differentiation ability when compared to their villous CTB counterparts.

To more closely evaluate the identity and differentiation potential of our TSCs, we next evaluated the expression of markers of villous CTB (EGFR and ITGA6), pcEVT (ITGA5 and ITGA2), and pan-EVT (HLA-G) in TSCs. We used six TSC lines derived in our lab from 6–8 week gestation placental tissues (including the 3 subjected to scRNA-seq above), performing flow cytometry for all 5 of these markers, and found these cells to almost uniformly express EGFR (95.5±3%), ITGA6 (94.6±2.4%), ITGA5 (98.6±0.8%), and ITGA2 (99.6±0.4%), with a small fraction co-expressing HLA-G (9.4 ±6.2%) (Fig. 4g). To test whether these markers are retained in TSCs following differentiation, we first performed directed differentiation of these cells into EVT or STB, using protocols described by Okae et al.[7] and performed immunofluorescent staining for ITGA5, along with other markers of differentiation (HLA-G for EVT, and SDC1 and CYP19A1 for STB). We noted that ITGA5 expression was lost following STB differentiation and retained when TSCs were differentiated into EVT (**Fig. S10a, S10b**). We also evaluated spontaneous differentiation potential of TSCs. We had previously shown that culture of first trimester CTB, plated on fibronectin in DMEM/F12+10% FBS media, allowed for differentiation of cells into either STB or EVT over 4 days, depending on oxygen tension (21% vs. 2%, respectively)[31]. Therefore, we similarly cultured TSCs for 4 days, and found that, by qPCR, they significantly upregulated STB markers, such as CGB, in normoxia and EVT markers, such as ASCL2, in hypoxia (Fig. 4h); interestingly, however, the EVT marker, ITGA1, was highest when the cells were cultured in normoxia. By morphology and staining, the TSCs formed large SDC1^+^ multinucleated cells in normoxia, but preferentially formed HLA-G^+^ mononuclear cells in hypoxia (**Fig. S10c**). These data suggest that, while first-trimester TSCs most closely resemble pcEVT, they do display bipotentiality during directed differentiation, although their bipotentiality is less clear following spontaneous differentiation.

### 2.5 Temporal analysis of TSC derivation shows gradual loss of CTB markers and selection for proximal column EVT markers

**Fig. 5:**
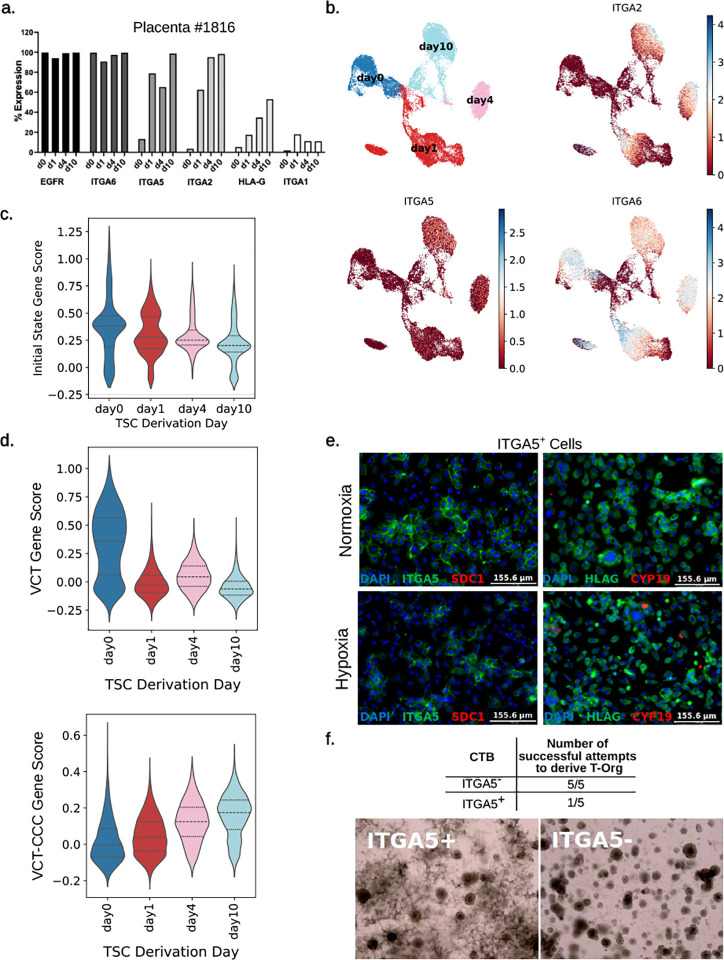
TSC diverge from vCTB and toward a pcEVT phenotype during *in vitro* derivation. (**a**) Percent of early gestation first trimester CTB expressing markers of CTB (EGFR, ITGA6), pcEVT (ITGA5, ITGA2), pan-EVT (HLA-G) and mature EVT (ITGA1), by flow cytometry, at 4 timepoints during TSC derivation. (**b**) UMAPs colored by day in TSC medium (top left) and expression of *ITGA2/5/6* in the same placenta as (a). (**c**) Violin plot showing initial state scoring of cells grouped by day of derivation in placenta #1816. Scoring was done using the significantly upregulated genes in initial state cells in first trimester placenta (Fig. 2b, **Table S10**). Dashed lines represent quartiles. (**d**) Violin plots, grouped by day of derivation, scoring cells from placenta #1816 based on the top 50 uniquely expressed genes as outlined by Arutyunyan et al.[8] for the cell types villous cytotrophoblast (VCT) and cytotrophoblast cell columns derived from VCT (VCT-CCC). Dashed lines represent quartiles. (**e**) Primary first trimester CTB, MACS-sorted based on ITGA5 expression, allowed to spontaneously differentiate over 4 days in either normoxia (21% oxygen) or hypoxia (2% oxygen), then fixed and stained for pcEVT marker ITGA5, the EVT marker HLA-G, STB markers SDC1, CYP19A1, and DAPI. Only ITGA5^+^ cells are shown, as ITGA5^−^ cells did not adhere to any substrate in 2D. (**f**) Organoid (T-Org) formation using first trimester CTB sorted for ITGA5. Table shows number of successful attempts to derive and culture organoids beyond 5 passages. Brightfield images of representative T-Org, derived from ITGA5^−^ and ITGA5^+^ CTB.

To determine how culture conditions affect the expression of CTB and pcEVT markers during TSC derivation, we isolated CTB from first-trimester placental tissues (**Table S1**) and evaluated them throughout a ten-day TSC derivation process in 2D (at isolation on day 0, and at days 1, 4, and 10, the last being 3 days after the first passage) using scRNA-seq and flow cytometry. We noted a fast rise in ITGA2 and ITGA5 expression by flow cytometry by day 1 of culture in TSC media (Fig. 5a); this was similarly noted at the RNA level for both of these genes, though it was more subtle for *ITGA5* (Fig. 5b). CTB markers, including *EGFR* and *ITGA6* remained the same (Fig. 5a, 5b). These results were confirmed in an additional placental sample using both scRNA-seq and flow cytometry (**Fig. S11a, S11b**). Next, to determine if our TSC derivation changed in initial state potential throughout the derivation process, we scored each day using the initial state upregulated genes from our integrated first trimester placentae. In both patients, the initial state score decreased over time and was lowest by day 10 of the derivation process (Fig. 5c, **S11c**). To identify the placental cell type most closely resembling cells emerging during TSC derivation, we once more evaluated both patient datasets using the top 50 cell type marker genes identified by Arutyunyan et al.[8]. Our data revealed a trend where exposure to TSC culture media corresponded to a decrease in VCT-associated gene expression and an increase in pcEVT-associated (VCT-CCC) gene expression (Fig. 5d, **S11d**). These results suggest that TSCs derived from CTB fraction of early first trimester (6–8 week gestational age) placentae quickly lose initial state CTB features and become more similar to EVT progenitor cells.

In order to compare TSCs to their most equivalent counterpart *in vivo*, we isolated CTB and MACS-sorted for ITGA5 in order to evaluate their differentiation potential. We used this marker instead of ITGA2, since, compared to ITGA2^+^ cells, ITGA5^+^ CTB comprised a larger proportion of isolated mononuclear trophoblast, thus providing sufficient cell numbers from one placenta for one experiment. We first plated the cells in 2D, and found that only ITGA5^+^ cells adhered to the tested substrates (Collagen IV or fibronectin), in both oxygen tensions tested (21% or 2%), in either TSC or DMEMF12/FBS media, while very few ITGA5^−^ cells did the same (**Fig. S11e**, and data not shown). In both oxygen tensions, after 4 days in culture, ITGA5^+^ cells upregulated the EVT marker, HLA-G, but not the STB markers, SDC1 or CYP19A1, while the few adhered ITGA5^−^ cells did not upregulate any markers (Fig. 5e, and data not shown). We next tested the ability of CTB, sorted for ITGA5, to be cultured in 3D and form organoids, and found that only ITGA5^−^ organoids showed the proper morphology and could be consistently maintained beyond 5 passages (Fig. 5f). The majority of the ITGA5^+^ organoids could not be maintained long-term and showed extensive outgrowth formation instead (Fig. 5f). These data show that ITGA5^+^ CTB lack bipotentiality and are unable to form organoids; instead, consistent with their EVT progenitor state, they are only able to spontaneously differentiate into HLA-G^+^ EVT.

## 3 Discussion

Defects in early placental development are thought to be at the root of common pregnancy disorders such as preeclampsia, fetal growth restriction, recurrent miscarriage, and stillbirth. Furthermore, through developmental programming, abnormal early placental development can impact the long-term health of the offspring[32]. Despite the critical role early placental development plays in both reproductive success and overall health, our understanding of the development of this transient but consequential organ remains elementary due to ethical constraints, the absence of comparable animal models, and, until recently, the lack of normal (non-transformed) *in vitro* cell models. The derivation of TSCs by Okae et al. in 2018 has truly transformed this field, allowing for probing of specific genes and pathways in trophoblast differentiation and function[7, 33–38]. However, recently, this model has come under significant scrutiny, particularly in comparison to the 3D trophoblast organoid (T-Org) models[15, 16] with respect to their differentiation potential[18, 33]. Here, we focused on 2D-cultured TSCs, derived from 6–8-week gestation placentae, and, using single-cell transcriptomics, comparing them to the heterogeneous population of CTB, in both early (6–8 week) and late (12–14 week) first trimester placentae, and within the basal (maternal) vs. chorionic (fetal) plates in order to identify the cell of origin for these stem cells.

We identified multiple CTB clusters in early and late first trimester placentae, which could be grouped into three broad CTB clades. Two of these (clades 1 and 2) primarily contained CTB from early first trimester, and one (clade 3) contained a much higher proportion of late first trimester CTB. Clade 3 showed enrichment in 10 genes involved in vascular remodeling, immune regulation, structural roles, and trophoblast differentiation, suggesting a role for these CTB in chorionic villous development, including angiogenesis, during this time period. Of the two early CTB clades, clade 2 cells lacked mature CTB markers and were enriched for immune response pathways, consistent with a protective barrier role for the implanting embryo[39, 40]. The other early CTB clade, clade 1, appeared to be more transcriptionally similar to the late CTB clade 3, and contained early bipotential progenitors. These “initial state” CTB in clade 1 expressed transcription factors known to regulate gene expression through epigenetic modification, such as *TET1* and *KMT2A*, as well as several cell cycle-related genes, including *HMMR* and *TROAP*, and were enriched in the basal plate, near the maternal surface. Although functional studies are needed to further characterize this cell population, these data suggest that bipotential CTB exist predominantly in early first trimester (6–8 weeks gestational age) and are enriched at/near the basal plate.

To better understand the differences in CTB composition in the basal plate (BP) and chorionic plate (CP), we performed spatial transcriptomics on early first trimester placentae. These two surfaces are known to perform very different tasks in the placenta, but interestingly, have both been suggested to harbor bipotential trophoblast stem cells[29, 41, 42]. Not surprisingly, we found significant transcriptional differences between CTB in these two compartments. Specifically, genes upregulated in the BP were enriched for the hypoxia and mTORC1 signaling pathway and highly expressed in our scRNA-seq EVT clusters, whereas the genes upregulated in the CP were enriched for the EMT pathway and more highly expressed in our pre-STB clusters. This suggested that there are important differences in differentiation potential between the CTB found within these two regions and prompted us to compare the CTB contained there at the single cell level. Interestingly, compared to CP-CTB, BP-CTB showed a higher initial-state cell score and had higher expression of the initial state marker *HMMR*, verified using *in situ* hybridization. These data indicate that CP-CTB are primarily a source of STB precursors, whereas the BP-CTB are more likely to form EVT but still hold the potential to differentiate into STB. In fact, studies using first trimester villous explant cultures have demonstrated the existence of two distinct CTB subtypes, with CTB at the villous tips (or anchoring columns at the basal plate) representing a more robust progenitor cells (e.g. more resistant to cell death *in vitro*), and able to give rise to EVT for many days in culture[40]. To our knowledge, ours is the first study to directly compare CTB from the basal and chorionic plates in first trimester placenta, using both spatial and single cell transcriptomics.

Recently, several groups have compared TSC to T-Org and *in vivo* trophoblast[8, 14, 18, 43], with a focus on TSC grown in 3D. Similar to our results, these studies have also concluded that the TSC state shares transcriptional hallmarks of proximal column, and not villous, CTB, thus predisposing differentiation towards EVT, not STB. Interestingly, we found that compared to the initial state CTB in early first trimester, our TSCs shared significant transcriptional overlap, including expression of the initial state markers *HMMR* and *TROAP*, and the canonical villous CTB markers *EGFR* and *ITGA6*. However, we also found large transcriptional differences between initial state CTB and TSC, with the latter showing upregulation of the cell motility-associated *CD9*, the versatile stem cell-associated marker *SOX9*, as well as the pcEVT-associated genes *ITGA2*, *ITGA5*, and *SOX15*; of note, *CD9, SOX9*, and *ITGA2* were also previously highlighted by Shannon et al. as markers of the TSC state[18, 44, 45]. At the same, TSCs also showed downregulation of numerous villous CTB genes, *BCAM, GPX3*, and *PEG10*, as well as *FOXP1, PBX1*, and *FOXO4*, which Shannon et al. identified as transcription factors driving CTB-specific gene regulons[18]. Another such gene, *PAGE4*, which we confirmed as a vCTB-specific gene by *in-situ* hybridization, is a stress-response gene with known anti-apoptotic roles in the prostate[46], but otherwise not well-characterized functionally. In fact, when we scored our integrated TSC and early first trimester trophoblast dataset, using markers from the recently published spatial transcriptomics study by Arutyunyan et al., we found that TSCs appeared to be most similar, not to vCTB, but to their proliferative EVT progenitor cell type VCT-CCC[8], equivalent to pcEVT. To confirm our scRNA-seq data, we characterized TSC lines from 6 different placentae, and demonstrated that, in fact, all lines uniformly expressed the pcEVT markers *ITGA2* and *ITGA5*. Notably, our TSCs retained the expression of *ITGA5*, only upon differentiation to EVT, but lost it with forskolin-induced differentiation into STB. As noted in previous reports, unlike trophoblast organoids, TSCs do not spontaneously form STB[14, 18]. In fact, when we allowed TSCs to spontaneously differentiate (by culture in DMEM/F12+FBS), the cells formed large syncytia that were positive for the STB marker SDC1, but also upregulated ITGA1, an EVT marker, in normoxia. ITGA1 has been noted to be increased in human pluripotent stem cell-derived syncytia, considered to be more representative of invasive primitive syncytium[47]. We therefore suggest that a more detailed evaluation of these TSC-derived multinucleated cells is needed, preferably by single nucleus RNA-seq, in comparison to *in vivo* STB and primitive syncytium, in order to identify their true cell identity.

It is possible that, similar to first trimester explant culture, where villous CTB quickly undergo cell death while basal plate CTB continue to give rise to EVT outgrowths[40], TSC culture conditions favor the stabilization and expansion of an EVT progenitor-like cell type, instead of a more uniformly bipotential vCTB-like cell-type. In fact, in our hands, first trimester CTB cultured in TSC media rapidly and significantly increased the expression of both ITGA2 and ITGA5, markers of pcEVT by flow cytometry. Our single cell transcriptomic analysis of TSC derivation from early first trimester CTB furthers this hypothesis, and suggests that culture in TSC media leads CTB to lose their initial state/transcriptional bipotentiality signature and gain a pcEVT-associated VCT-CCC signature, within the first 10 days of the TSC derivation process. Furthermore, when we sorted our TSC based on ITGA5 expression, only ITGA5^+^ CTB adhered to matrix-coated plates in 2D, spontaneously differentiating into only EVT-like cells, while ITGA5^−^ cells, could only be cultured in 3D, as T-Org. These data further support the hypotheses put forth above, that TSCs are not only biased toward the EVT lineage, but given the lack of ability to spontaneously form *bona fide* STB, may therefore not be an appropriate model for true STB differentiation. Instead, T-Org consist of cells that better model villous CTB and spontaneously form an inner STB layer[14, 18]. Nevertheless, single nucleus RNA-seq analysis of multinucleated cells, derived from TSC (cultured in 2D or 3D) vs. T-Org, and comparison to *in vivo* STB and primitive syncytium, are needed, in order to characterize the identity of multinucleated cells across these models.

In conclusion, our findings offer an in-depth transcriptomic perspective of the first trimester placenta and have significant implications for understanding early developmental stages of this organ, as well as the utility of TSC as a model system. The identification of distinct CTB populations, especially those located at the basal and chorionic plate, and their developmental trajectories provide a valuable resource for future studies of distinct subtypes of first trimester CTB. The integration of TSC data, with its similarities and differences to *in vivo* CTB, highlights the potential and limitations of TSCs in recapitulating early trophoblast differentiation. Finally, validation of scRNA-seq data, not just with spatial transcriptomics and *in-situ* hybridization, but also with primary cell isolation and culture, offers further insights into the actual differentiation potential and functions of distinct CTB progenitor states. Future studies should aim to further elucidate the factors influencing TSC culture and differentiation and develop conditions for culture and characterization of other CTB progenitors within early gestation human placentae.

### 3.1 Limitations of the study

The 10X Chromium platform used in this study is limited in the size of cell it is able to capture. While differential recovery is not considered an issue for cells below 30 *μ*m in this system, this has not been tested for cells above this size. This most likely excluded capture of mature multinucleated STB and trophoblast giant cells. Moreover, our dataset was comprised of cells from a relatively small number of presumably normal placentas, sequenced at a relatively shallow read-depth compared to bulk RNA-seq, potentially constraining our ability to find important but lowly-expressed genes. Finally, trajectory analysis using RNA velocity relies on static snapshots of cellular states at the moment of measurement, and is therefore reliant on only a small number of genes, which appear to obey the simple interpretable kinetics used by RNA velocity[48]. Future studies should use single nucleus RNA-seq, combined with metabolic labeling, and compare 2D and 3D models of trophoblast to multinucleated cells within normal tissue, in order to identify the optimal ways to model these mature cell types.

## 4 Methods

### 4.1 Patient recruitment and tissue collection

Human placental tissue samples for this study were collected under a UC San Diego Institutional Review Board-approved protocol; all patients gave informed consent for collection and use of these tissues. All samples were collected from pregnant individuals, undergoing elective termination of pregnancy, providing written informed consent.

Early first trimester placental tissues, including those that were used to derive TSCs and organoids, were taken from patients with gestational ages ranging from 5 weeks and 5 days to 8 weeks and 4 days. Late first trimester placental tissues (n=2) were taken from patients with gestational ages of 12 weeks and 2 days and 13 weeks (**Table S1**). Gestational age was determined based on crown-rump length, measured on ultrasound, and was stated in weeks/days from the first day of the last menstrual period. “Normal” sample was defined as a singleton pregnancy without any detectable fetal abnormalities on ultrasound.

The biological sex of the placental tissues used in this manuscript was not considered in the analyses presented here. The small sample size used in this study prohibited the authors from making meaningful inferences about biological sex differences. Sex determination can be inferred by those using the data in this study by expression of Y chromosome-linked genes.

### 4.2 Trophoblast sample collection and preparation

Samples were processed within 2 hours of collection according to our established protocol[30]. For whole placental samples, including TSC and organoid derivation, chorionic villi were minced and subjected to three sequential enzymatic digestions with Trypsin and DNase, followed by Percoll (Sigma-Aldrich) gradient separation. For single cell analysis, after filtration, cells were counted and loaded on the 10x Genomics chip. For basal and chorionic sample collection, we collected the villous tips (basal fraction) and the gestational sac (chorionic fraction) under a dissecting microscope, while the villi in the middle section were discarded. The two fractions were subjected to the same sequential enzymatic digestion and after filtration cells were separated on a Percoll (Sigma-Aldrich) gradient. Cell suspensions were loaded onto the 10x Genomics Chromium Controller for GEM generation. All single-cell libraries were constructed using the Chromium Next GEM Single Cell 3, Library and Gel Bead Kit v2, v3, and v4 (10x Genomics), following the manufacturer’s protocol for all steps. Single-cell libraries were sequenced on an Illumina NovaSeq 6000 instrument. Capture rate and sequencing depth can be found in **Table S1**. scRNA-seq datasets are publicly available on the GEO repository (GSE270174). Leftover cells were lysed for RNA isolation using NucleoSpin kit (Macherey-Nagel) and proper basal vs. chorion separation was verified by enrichment of *CDX2* (chorionic) and *ASCL2* (basal) expression by qPCR. Libraries were prepared only from sample pairs showing correct marker enrichment.

### 4.3 Patient-derived trophoblast stem cell establishment, culture, and differentiation to EVT and STB

Following Percoll (Sigma-Aldrich) separation, first trimester (GA 6–8 weeks; n=8) CTB were MACS-purified with either a PE-conjugated anti-ITGA6 antibody (Biolegend #313612) or an APC-conjugated anti-EGFR antibody (Biolegend #352906). MACS-purified CTB were then plated for TSC derivation as previously described[9, 13] and cultured with modified TSC media[49]. Once cell morphology had stabilized (after 6–8 passages), TSCs were evaluated for expression of various surface markers, including EGFR, HLAG, and a panel of integrins by flow cytometry (see below).

Directed EVT differentiation was performed by plating 75,000 cells/well onto a 6-well plate pre-coated with 20*μ*g/ml fibronectin in EVT differentiation media as described by Okae et al.[7]. On day 3, EVT medium was replaced without NRG1, and Matrigel concentration was reduced to 0.5% until day 5. On day 5, cells were re-plated onto a 6-well plate pre-coated with 20*μ*g/ml fibronectin using the latter media (without NRG1 and with reduced Matrigel). For immunostaining, cells were fixed on day 6.

Directed STB differentiation was performed by plating 25,000 cells/well onto a 6well plate pre-coated with 2.5*μ*g/ml collagen IV in the STB differentiation media as described by Okae et al.[7]. Cells were fixed on day 6 for immunostaining.

For spontaneous differentiation, two TSC lines (1000P and 1048P) were plated at a density of 100,000 cells/well in a 12-well plate coated with 20*μ*g/ml fibronectin and cultured in Dulbecco’s modified Eagle’s medium/F12 with 10% FBS under normoxia (21% O2) or hypoxia (2% O2, using an XVIVO X3 Workstation). After 4 days, cells were fixed for immunostaining, or collected for RNA isolation and qRT-PCR.

### 4.4 Isolation and plating of first trimester cytotrophoblasts

CTB were isolated from the first trimester placental tissues using sequential digestion with Trypsin and DNase, followed by Percoll (Sigma-Aldrich) gradient separation as previously described[50]. For the plating experiment, one million cells were plated per well in a 6-well plate, coated with 20*μ*g/ml fibronectin, and cultured in Dulbecco’s modified Eagle’s medium/F12 with 10% FBS, 1x penicillin/streptomycin, and 50*μ*g/ml gentamicin as previously described[50].

In a separate set of experiments, CTB were sorted for ITGA5 by magnetic-activated cell sorting (MACS), following the manufacturer’s protocol (Miltenyi). ITGA5 positive and negative cells were then plated onto 20*μ*g/ml fibronectin-coated plates and cultured again in DMEM/F12 with 10% FBS and antibiotics under normoxia (21% O2) or hypoxia (2% O2, using an XVIVO X3 Workstation). Cells were then fixed for immunostaining.

### 4.5 Trophoblast organoid derivation and culture

CTBs were isolated from first trimester placental tissues using sequential digestion with Trypsin and DNase, followed by Percoll (Sigma-Aldrich) gradient separation as previously described[50]. Isolated trophoblast cells were sorted for ITGA5 by MACS with ITGA5-FITC antibody (Biolegend #328008), following the manufacturer’s protocol (Miltenyi). ITGA5 positive and negative cells were counted and plated into Phenol Red free Matrigel (Corning) domes at 37,000 cells/15*μ*l domes in trophoblast organoid media (TOM) comprised of Advanced DMEM/F12 (Life Technologies), 1x N2 and 1x B27 supplements (Life Technologies), 2mM Glutamax (Life Technologies), 1.25mM N-Acetyl-L-cysteine (Sigma), 500nM A83–01 (Tocris), 1.5*μ*M CHIR99021 (Sigma-Aldrich), 80ng/mL human R-spondin 1 (Peprotech), 50ng/mL human EGF (R&D), 50ng/mL human HGF (Stemcell Technologies), 2.5*μ*M prostaglandin E2 (PGE2) (Selleck Chemicals), 100ng/mL human FGF2 (BioPioneer), and 2*μ*M Y-27632 (Selleck)[15]. Cultures were maintained in a 37°C humidified incubator with 5% CO2 and media changed every 2–3 days. After 10–12 days, organoids were passaged following mechanical dissociation as described in Turco et al.[15]. After the first passage, organoids were maintained in TOM media and passaged with a combination of TryPLE Express (Thermo Scientific) treatment for 3–5 min. followed by mechanical dissociation every 10–14 days and re-seeded at 20,000 cells/20*μ*L in Matrigel dome[51].

### 4.6 Flow cytometry analysis

Isolated CTB and derived TSCs were stained, either individually with EGFR-APC (Biolegend #352906), HLAG-PE (ExBIO #1P-292-C100), ITGA6-AF647 (Biolegend #313610), ITGA5-FITC (Biolegend #328008) and ITGA2-AF594 (R&D #FAB1233T), or using a multi-color panel of EGFR-PE/Cy7 (Biolegend #352910), HLAG-APC (ExBIO #1A-292-C100), ITGA1-PE (Biolegend #328304), ITGA2-AF700 (R&D #FAB1233N), ITGA5-FITC (Biolegend #328008) and ITGA6-BV421 (Biolegend #313624), with relative isotype control antibodies, for 1 hour in flow cytometer wash buffer (1% FBS, 2%BSA and 0.03% Sodium Azide in PBS) in the dark. After staining, cells were washed three times with wash buffer and fixed with 2% PFA, and samples were acquired on either a FortessaX20 or Fortessa (BDLSR-Fortessa) flow cytometer (BD Biosciences). Data analysis was performed using FlowJo and data were represented as bar graphs using GraphPad Prism.

### 4.7 Immunofluorescent staining of cells and tissues

Cells were fixed with 4% paraformaldehyde (PFA) in PBS for 10 minutes, washed once with PBS, then permeabilized with 0.1%Triton X for 10 mins at room temperature (RT), and washed three times with PBS. Cells were blocked with 5% Goat serum and 0.05% Triton X in PBS for 1 hour at RT. Cells were stained overnight at 4°C with primary antibodies, including mouse anti-SDC1 (0.5*μ*g/ml, Abcam #ab34164), rabbit anti-ITGA5 (1.1*μ*g/ml, Abcam, #ab150361), and mouse anti-CYP19A1 (1.0*μ*g/ml, Novus Biologicals, #NBP3–07826), and Rabbit anti-HLAG (0.5*μ*g/ml, Abcam #ab283260), followed by secondary antibodies (Alexa Fluorconjugated goat anti-rabbit or anti-mouse antibodies, Thermo Fisher Scientific) for 1 hour at RT in the dark. Nuclei were stained with DAPI, and images were taken on a Leica fluorescence microscope.

Immunofluorescent staining was also performed on formalin-fixed paraffin-embedded (FFPE) placental tissues. 5-mm serial sections were cut, dried, and deparaffinized and rehydrated using xylene and ethanol. Sections were permeabilized in a 0.1% triton solution in 1x TBS for 10 minutes, then subjected to antigen retrieval in EDTA at 100°C for 15 minutes. Tissue sections were blocked using 10% normal goat serum (Jackson ImmunoResearch, Cat# 005-000-001) and 5% BSA (Gemini BioProducts, Cat#700–110) in TBST for 30 minutes at 37°C. Serial sections were stained with mouse anti-HLAG (1.0*μ*g/mL, Abcam, #ab283270), rabbit anti-ITGA2 (1.0*μ*g/mL, Bioss, #bsm-52613R) and rabbit anti-ITGA5 (1.1*μ*g/ml, Abcam, #ab150361) and incubated at 4°C overnight. The following day, slides were washed with 1x TBS then incubated with Alexa Fluor 488 and 594 conjugated goat anti-rabbit and goat antimouse (Thermo Fisher) for 1 hour at room temperature covered from light. Slides were then washed in a 1:5000 dilution of DAPI in 1x TBS for 7 minutes, washed in 1x TBS, then mounted using Prolong Gold (Invitrogen). Images were acquired using a Leica fluorescence microscope and individual channel photos were merged using ImageJ.

### 4.8 *In situ* hybridization

First trimester placental tissue samples were fixed in a neutral-buffered formalin and embedded in paraffin wax. Five *μ*m sections were subjected to *in-situ* hybridization on a Ventana Discovery Ultra automated stainer (Ventana Medical Systems) at the UC San Diego Advanced Tissue Technology Core laboratory. RNAscope probes specific to human *PAGE4, HMMR, TROAP, ITGA5*, and *ITGA2* were purchased from ACD-Bio. Following amplification steps, the probes were visualized using an HRP-based reaction, visualizing single probes (*PAGE4, HMMR*, and *TROAP*) in brown and dual probes in teal (*ITGA5*) and red (*ITGA2*); slides were counterstained with hematoxylin. Slides were visualized by conventional light microscopy on an Olympus BX43 microscope (Olympus).

### 4.9 GeoMx digital spatial profiler whole transcriptome assay

For spatial transcriptomic analysis, three 6-week gestation placentas, previously formalin-fixed and paraffin-embedded, were selected from the Center for Perinatal Discovery’s biorepository (*Table S1*). Samples containing a visible, relatively intact embryonic sac were selected so that the basal and chorionic areas were easily identifiable. 5*μ*m sections were mounted on SuperFrostTM Plus slides and used within 2 weeks. Sections were deparaffinized and incubated overnight with the Whole Transcriptomic Assay UV-cleavable biological probes according to manufacturer’s instructions. Samples were stained with CONFIRM^®^ anti-EGFR (5B7) Rabbit Monoclonal Primary Antibody (Ventana #790–4347, 1:20 dilution) and anti-HLA-G (4H48) mouse primary antibody (Abcam #ab52455, 1:1000 dilution) for 2 hours followed by secondary staining for 1h with Alexa Fluor anti-rabbit-647 (Life Technologies, 1:500 dilution), Alexa Fluor anti-mouse-594 (Life Technologies, 1:500 dilution), and SYTO13 (Nanostring, 1:10 dilution) for nuclei staining. Slides were scanned on a GeoMx digital spatial profiler (Nanostring). Based on the morphology markers, regions of interest (ROIs) containing at least 100 EGFR^+^/HLA-G^−^ nuclei were manually selected. In total, we identified 29 ROIs, 14 from the basal plate and 15 from the chorionic plate. Samples were collected and libraries prepared according to the manufacturer’s instructions. Libraries were pair-end sequenced on a NovaSeq 6000 in the UCSD IGM core at 3 million reads per sample. Fastq files were converted into DCC files following the GeoMx NGS pipeline (v.2.0.0.16). Data QC was performed on the GeoMx DSP software according to standard parameters (minimum 100 nuclei per ROI, area size at 1000*μ*m2, negative probe count to 1, sequencing saturation at 30, Q3 normalization) and then exported for further downstream analysis. Genes were considered differentially expressed between the basal and chorionic plate using Mann-Whitney U test (adj. p-value < 0.05 and log fold change > 1), (**Table S11**). Data are publicly available on the GEO repository (GSE270174).

### 4.10 RNA isolation and qRT-PCR

On day 4, cells were collected, and RNA isolated using NucleoSpin^®^ (Macherey-Nagel, USA) kit. 300ng of RNA was reverse transcribed to prepare cDNA using PrimeScript^™^ RT reagent kit (TAKARA, USA) following the manufacturer’s instructions. qRT-PCR was performed using Power SYBR^®^ Green RT-PCR Reagents Kit (Applied Biosystems, USA) using the following primer pairs in a 5’ to 3’ orientation: ASCL2 forward CACTGCTGGCAAACGGAGAC, ASCL2 reverse AAAACTCCAGATAGTGGGGGC; CGB forward ACCCTGGCTGTGGAGAAGG, CGB reverse ATGGACTCGAAGCGCACA; ITGA1 forward CTGGACATAGTCATAGTGCTGGA, ITGA1 reverse ACCTGTGTCTGTTTAGGACCA; L19 forward AAAACAAGCGGATTCTCATGGA, L19 reverse TGCGTGCTTCCTTGGTCTTAG. Data were normalized to L19, analyzed according to the delta Ct method and shown as fold-change over undifferentiated TSC. GraphPad Prism was used to perform statistical analysis. Ordinary One-way ANOVA with multiple comparison was used to calculate the statistical analysis related to with undifferentiated TSCs. Data is expressed as mean ± standard deviation of 2^−ddCt^ values. The level of statistical significance was set at p < 0.05.

### 4.11 Trophoblast scRNA-seq library construction and sequencing

Cells were run on the 10X Genomics platform with the Chromium Next GEM Single Cell 3’ kit. Samples and libraries were prepared following the manufacturer’s protocol to attain 5,000 to 10,000 cells per reaction. Libraries were pooled and sequenced using the Illumina NovaSeq 6000 sequencer. Capture rate and sequencing depth can be found in **Table S1**.

### 4.12 Single-cell RNA-seq data analysis

#### 4.12.1 Data mapping and technical artifact removal

All scRNA-seq data was mapped using the STARsolo function of STAR (v.2.7.10a)[52]. STARsolo performed error correction and demultiplexing of cell barcodes using user-input whitelist (3M-3pgex-may-2023.txt for version 4, 3M-february-2018.txt for version 3, and 737K-august-2016.txt for version 2 of the 10X Genomics Chromium System), mapped reads to the reference genome (GRCh38, version 32, Ensembl 98), deduplicated and error corrected UMIs, quantified per-cell gene expression, and spliced and unspliced reads similar to Velocyto. The following are the CellRanger mimicking commands used: –outFilterScoreMin 30 –readFilesCommand zcat –soloCBmatchWLtype 1MM_multi_Nbase_pseudocounts –soloUMIfiltering MultiGeneUMI_CR –soloUMIdedup 1MM_CR –soloCellFilter EmptyDrops_CR –soloUMIlen 12 –soloType CB_UMI_Simple –soloCBwhitelist 3M-february-2018.txt –clipAdapterType CellRanger4 –outFileNamePrefix name –outSAMattributes All –outSAMtype BAM SortedByCoordinate –quantMode GeneCounts –soloCBlen 16 –soloCBstart 1 –soloUMIstart 17 –soloFeatures Gene GeneFull SJ Velocyto. Following STARsolo quantification, the data was run through CellBender’s (v. 0.3.0) ‘remove-background’ command[19] to remove systematic biases and background noise due to ambient RNA molecules and random barcode swapping.

#### 4.12.2 Data pre-processing, filtering, integration, and quality control

The data was then loaded into Scanpy (v. 1.10.1)[22] for preprocessing, filtering, and quality control. Doublet removal was performed by Scrublet[20] with an expected doublet rate of 0.076. Following doublet filtering, cells with less than 200 genes expressed were removed and genes detected in less than 3 cells were removed. Next, cells with greater than 20% mitochondrial DNA content were removed as well as cells with greater than 5% ribosomal gene expression or 15% hemoglobin gene expression. The data was then normalized, and the cell cycle was regressed out using the cell cycle genes from Macosko et al.[53], and highly variable genes were selected. If data was specified as being integrated, it was done using scvi-tools (v. 1.1.2)[21], specifically using the scVI (single-cell Variational Inference) model with “batch” and “cell source” as the categorical covariate keys. Integration was performed before clustering but after combining all datasets being analyzed and performing the above quality control steps.

#### 4.12.3 Clustering and clustering annotation

Integrated datasets were clustered using Scanpy’s Leiden clustering algorithm, an improved version of the Louvain algorithm[54], with a resolution of 0.75 unless otherwise specified. Marker genes were calculated using default parameters and clusters were annotated as trophoblast if they expressed all or some of the genes *KRT7, GATA3, PAGE4, TFAP2A, HLA-G*, or *CYP19A1* and lacked all or most of the expression of the genes *HLA-A, HLA-B, HLA-DRA, CD14, VIM*, or *CD34*. The non-trophoblast clusters were then removed from downstream analyses and the remaining trophoblast clusters were re-clustered using the same resolution after the nearest neighbors distance was re-calculated and embedded using UMAP. Trophoblast clusters were annotated using the following gene expression: CTB (*BCAM, SLC6A4, PARP1, EGFR, TP63, SLC27A2*, and *TENM3*); EVT (*HLA-G, ASCL2, ITGA5, FN1, LAIR2, NOTUM, LPCAT1, PRG2*, and *AOC1*); Pre-STB (*CYP19A1, ERVV-1, GREM2, ERVFRD-1, LGALS16, EPS8L1*, and *SERPINB2*). Hierarchical clustering using Pearson correlation method with complete linkage was used to assess the relative distance between clusters. Differential expression between two groups was performed using ‘rank_genes_groups’ and setting one cluster as the reference and the other as the ‘group’. Genes were considered up- or downregulated using a log2 fold change of 1.5 unless otherwise stated.

#### 4.12.4 Trajectory inference and initial state determination

Trajectory inference was performed using scVelo (v. 0.3.2), an RNA velocity[55] analysis toolkit for single cells that leverages splicing kinetics to recover directed dynamic information using an expectation-maximization framework[23] or a deep generative model[56]. A function from https://github.com/JBreunig was used to put together all the spliced and unspliced data before preprocessing and moments calculations were performed. Velocities were estimated in a gene-specific manner using the stochastic mode for all trajectories except for the individual patient trajectories, which used the dynamical mode.

To compute the initial state, we used CellRank2 (v. 2.0.4)[24], a Markov state modeling framework for cellular dynamics, and the velocity kernel, which computes a transition matrix based on RNA velocity, along with the Generalized Perron Cluster Analysis algorithm (GPCCA estimator)[25]. When computing the initial state for the dataset containing the combined early- and late placental samples, first the velocity kernel and connectivity kernel were combined in a 0.8/0.2 ratio. Then using the GPCCA estimator, the terminal states were set to the EVT and pre-STB clusters and then the number of states was selected to be four. From these four states, the initial state was predicted.

### 4.13 Quantification and Statistical Analysis

Data are reported as mean ± standard deviation. Statistical analyses were performed using GraphPad Prism. Ordinary one-way ANOVA and multiple comparison corrections were used to calculate the statistical significance compared to undifferentiated TSC. qPCR data were analyzed following the ddCt method: data were normalized on the housekeeping gene L19 and expressed as fold change (2^−ddCt^) over undifferentiated TSC. The level of statistical significance was set at p < 0.05. For scRNA-seq statistical analyses, please refer to the applicable section in the methods details.

### 4.14 Resource Availability

#### 4.14.1 Materials availability

This study did not generate new unique reagents. Requests for further information, reagents, and human trophoblast stem cell lines used in this study should be directed and will be fulfilled by the Lead contact, Mana Parast (mparast@health.ucsd.edu).

#### 4.14.2 Data and code availability

- Single-cell RNA-seq data and GeoMx digital spatial profiler data have been deposited at GEO and are publicly available as of the date of publication. Accession numbers are listed in the key resources table (GSE270174). Microscopy data reported in this paper will be shared by the lead contact upon request.
- This paper does not report original code. The code used for data processing and analysis has been deposited at Zenodo and is publicly available as of the date of publication.
- Any additional information required to reanalyze the data reported in this paper is available from the lead contact upon request.

## Supplementary Files

This is a list of supplementary files associated with this preprint. Click to download.

- tablescombinedJune25.xlsx
- submissionJune25supplementaryfigures.pdf

Supplementary Figures

**Figure S1: First trimester placental single cell RNA-seq: Integration UMAPs.** (**a**) UMAPs of placental samples before integration colored by the patient number (left) and batch number (right). (**b**) UMAPs of placental samples after integration colored by the patient number (left) and batch number (right).

**Figure S2: First trimester placental single cell RNA-seq: Removal of non-trophoblast clusters and reclustering of trophoblast cells.** (**a**) UMAP following the removal of clusters in Fig. 1a deemed to be non-trophoblast cells. (**b**) Reclustering of cells after removal of most of the non-trophoblast cells. (**c**) Dot plot and hierarchical clustering showing expression of trophoblast- and non-trophoblast-associated genes in reclustered UMAP shown in (b). (**d**) Dendrogram showing the transcriptional similarity between clusters in UMAP shown in (b), and annotated using dotplot shown in Fig. 1c, with clades colored by the cluster color of UMAP in Fig. 1d following the combining of clusters.

**Figure S3: Upregulated transcription factors in initial state cells.** (**a**) UMAP showing the expression of the transcription factors MXD3 and CENPA that were differentially upregulated (adj. p-value < 0.05 and log fold change > 1) in the initial state cells (n=30), compared to the rest of the trophoblast cells in our integrated first trimester trophoblast dataset (**Table S10**), and were part of the CTB1 rank velocity genes (**Table S9**). (**b**) Dot plot showing the mean cluster expression and the fraction of cells in each cluster expressing differentially upregulated transcription factors (adj. p-value < 0.05 and log fold change > 1) in the initial state cells (n=30) compared to the rest of the trophoblast cells in our integrated first trimester trophoblast dataset (**Table S10**), and were part of the CTB1 rank velocity genes (**Table S9**).

**Figure S4: Spatial transcriptomic profiling of basal and chorionic plate CTB.** (**a**) Representative scan of formalin-fixed paraffin-embedded placental tissue (placenta # 1459P) used for GeoMx-based digital spatial profiling, showing tissue stained for EGFR to identify vCTB, with selection of Regions of Interest (ROI’s) near the basal (BP, arrowheads) and chorionic plate (CP, arrows) based on spatial relation to the gestational sac. (**b**) Heatmap displaying the expression of genes that were determined to be differentially expressed (adj. P-value < 0.05 and log fold change > 1) between BP and CP CTB in 29 ROI’s, located within three different placentae, using the GeoMx whole transcription atlas (WTA) panel. (**c**) UMAPs of early and late first trimester integrated scRNA-seq data, scored using the genes upregulated in the basal plate (top) or the chorionic plate (bottom), based on the GeoMx WTA data.

**Figure S5: Single cell RNA-seq of basal and chorionic plate CTB.** (**a**) QPCR showing the relative expression of the chorionic plate marker CDX2 and the basal plate marker ASCL2 in cells separated into basal plate (BP, dark blue) and chorionic plate (CP, light blue) fractions. Single-cell RNA-seq was then subsequently performed on these fractions. (**b**) UMAP showing the clustering of the basal and chorionic plate scRNA-seq dataset (left) and dot plot showing the mean expression and fraction of cells expressing various trophoblast (grey) and non-trophoblast (light blue) marker genes (right). (**c**) Dot plot and hierarchical clustering of cell clusters in the BP and CP dataset, showing the mean expression and fraction of cells expressing trophoblast cell type-specific markers (CTB in grey, EVT in yellow, and pre-STB in green).

**Figure S6: Basal and chorionic plate cell scoring using spatial transcriptomics data.** (**a**) Violin plot showing the basal and chorionic plate cells, scored using the genes found to be differentially upregulated in the basal plate regions of interest (ROIs) compared to the chorionic plate ROIs from the spatial transcriptomic dataset. (**b**) Violin plot showing the basal plate and chorionic plate cells scored using the genes found to be differentially upregulated in the chorionic plate ROIs compared to the basal plate ROIs from the spatial transcriptomic dataset. Dashed lines indicate quartiles and * indicates Wilcoxon rank-sum p-value < 0.001.

**Figure S7: Expression of initial state marker genes in the basal and chorionic plate.** (**a**) UMAPs showing the expression of initial state marker genes *HMMR* and *TROAP* in basal and chorionic plate scRNA-seq dataset. Cluster CTB1 is marked by a dotted circle. (**b**) UMAPs showing the expression of initial state transcription factors *MXD3* and *CENPA* in basal and chorionic plate scRNA-seq dataset. Cluster CTB1 is marked by a dotted circle. (**c**) *In-situ* hybridization of the initial state marker, HMMR, in a 6-week placenta (same one shown in Fig. 2d), comparing expression at/near basal vs. chorionic plate. Villous CTB in chorionic villi (“v”) are marked by arrowheads. The gestational sac is labeled “sac,” while the proximal column trophoblast are marked by a circle. Scale bar=125 *μ*m.

**Figure S8: Early first trimester and trophoblast stem cell (TSC) scRNAseq dataset, following integration, clustering, and annotation.** (**a**) UMAP of all cells from early first trimester placenta and TSC, following quality control filtering, integration, and clustering. (**b**) Dot plot and hierarchical clustering of cell clusters using trophoblast and non-trophoblast specific gene expression. (**c**) Dot plot and hierarchical clustering of cell clusters using cell type specific gene expression. (**d**) Hierarchical clustering dendrogram showing the transcriptomic similarity (Pearson correlation) between cell clusters (left), UMAP of clusters annotated based on cell type specific gene expression shown in part (c) (middle) and UMAP showing the location of TSC (light blue) vs. first trimester placental cells (dark blue) (right).

**Figure S9: Differences in gene expression between TSC and initial state CTB.** (**a**) UMAPs showing the expression of genes upregulated in TSCs compared to initial state CTB. (**b**) UMAPs showing the expression of genes upregulated in initial state CTB compared to TSCs. (**c-d**) Immunohistochemistry of first trimester placenta with antibodies against ITGA5 and ITGA2, co-stained either with HLA-G (**c**) or CYP19A1 (**d**). ITGA5 is only expressed in column trophoblast, while ITGA2 is expressed in rare proximal column trophoblast (arrow in panel C), but also in fetal endothelial cells (panel D). Scale bar=156 *μ*m. (**e**) *In-situ* hybridization of first trimester placenta with probes against *ITGA5* (teal) and *ITGA2* (magenta). Rare dual-positive cells are noted in the proximal columns (arrowheads). V=villous core; CC=cell column. Scale bar=62.5 *μ*m.

**Figure S10: Directed and spontaneous differentiation of TSC.** (**a**) Immunohistochemistry of STB markers (SDC1 and CYP19A1), pcEVT marker (ITGA5), and EVT marker (HLA-G) following directed differentiation of TSCs to EVT. (**b**) Immunohistochemistry of STB markers (SDC1 and CYP19A1), pcEVT marker (ITGA5), and EVT marker (HLA-G) following directed differentiation of TSC to STB. (**c**) Immunohistochemistry of STB marker (SDC1) and EVT marker (HLA-G) following spontaneous differentiation of TSC (cultured in DMEM-F12+FBS in either normoxia/21% oxygen or hypoxia/2% oxygen). By morphology and staining, the TSCs formed large SDC1^+^ multinucleated cells in normoxia, but preferentially formed HLA-G^+^ mononuclear cells in hypoxia.

**Figure S11: Derivation of TSC from early first trimester CTB shows gradual progression toward a pcEVT phenotype.** (**a**) Boxplots showing expression of *ITGA2, ITGA5*, and *ITGA6*, based on scRNA-seq of CTB (isolated from placenta #1815), cultured in TSC derivation media. (**b**) Percent of early gestation first trimester CTB expressing markers of CTB (EGFR, ITGA6), pcEVT (ITGA5, ITGA2), pan-EVT (HLA-G) and mature EVT (ITGA1), by flow cytometry, at 4 timepoints during TSC derivation, in placenta #1815. (**c**) Violin plot showing initial state scoring of cells grouped by day of derivation, in placenta #1815. Scoring was done using the significantly upregulated genes in initial state cells in first trimester placenta (Fig. 2b, **Table S10**). Dashed lines represent quartiles. (**d**) Violin plots grouped by day of derivation, scoring cells from placenta #1815, based on the top 50 uniquely expressed genes as outlined by Arutyunyan et al.[8], for the cell types villous cytotrophoblast (VCT) and cytotrophoblast cell columns derived from VCT (VCT-CCC). Dashed lines represent quartiles. (**e**) Isolated CTB and MACS-sorted for ITGA5 cells plated in 2D. Pictures show that only ITGA5^+^ cells adhered to the tested substrates (fibronectin), in both oxygen tensions tested (21% or 2%, 21% shown), in TSC media, while very few ITGA5^−^ cells did the same.

Supplementary Tables

**Table S1:** Sample and sequencing metrics.

**Table S2:** Top 200 marker genes in each cluster in the first trimester placenta samples.

**Table S3:** Marker genes after removal of non-trophoblast clusters, reclustering, and annotation of clusters.

**Table S4:** Cell numbers of each cluster by gestational age for UMAP shown in **Fig. S2d**.

**Table S5:** Top 200 marker genes in each cluster of the merged primary placenta dataset shown in Fig. 1d.

**Table S6:** Genes upregulated (adj. p-value < 0.05 and log fold change > 1) in CTB3 (primarily composed of late first trimester CTB) vs CTB1 and CTB2 (primarily composed of early first trimester CTB) (Top) and genes upregulated (> 1 Log2 fold change and adj. p-value < 0.05) in CTB1 and CTB2 (primarily composed of early first trimester CTB) vs CTB3 (primarily composed of late first trimester CTB) (Bottom).

**Table S7:** Genes upregulated (> 1.5 Log2 fold change and adj. p-value < 0.05) in CTB1 vs CTB2 (both primarily composed of early first trimester CTB).

**Table S8**: Genes upregulated (> 1.5 Log2 fold change and adj. p-value < 0.05) in CTB2 vs CTB3 (both primarily composed of early first trimester CTB).

**Table S9:** Genes that best explain the RNA velocity vector field in Fig. 2a. These genes, called rank velocity genes, may play a role in driving the differentiation of CTB. Rank velocity genes with a minimum r^2^ value of 0.5 and a minimum correlation coefficient between spliced and unspliced genes of 0.3 by cluster.

**Table S10**: Genes upregulated (adj. p-value < 0.05 and log fold change > 1) in initial state cells (n=30) vs all other cells in the early and late first trimester integrated placental dataset.

**Table S11:** Genes upregulated (adj. p-value < 0.05 and log fold change > 1) in either the basal plate ROIs or chorionic plate ROIs in GeoMx spatial WTA dataset.

**Table S12:** Top 200 marker genes of all clusters after integration of early first trimester trophoblast and TSC single cell data (Fig. 4a).

**Table S13:** Genes upregulated (adj. p-value ¡ 0.05 and log fold change ¿ 1) in initial state cells (n=30) vs TSC, in the early first trimester and TSC integrated dataset (Fig. 4a).
